# Altered follicular helper T cell impaired antibody production in a murine model of myelodysplastic syndromes

**DOI:** 10.18632/oncotarget.21548

**Published:** 2017-10-06

**Authors:** Huijuan Jiang, Ningbo Cui, Liyan Yang, Chunyan Liu, Lanzhu Yue, Lifang Guo, Huaquan Wang, Zonghong Shao

**Affiliations:** ^1^ Department of Hematology, General Hospital, Tianjin Medical University, Tianjin 300052, China

**Keywords:** myelodysplastic syndromes, follicular helper T cell, CXCR5, PD-1, ICOS

## Abstract

Myelodysplastic syndromes (MDS) are a group of clonal hematopoietic diseases which have a high risk of progressing to acute myeloid leukemia. MDS patients have immunologic deficiency, including T and B cells dysfunction. Follicular T helper cells (Tfh, CD4^+^CXCR5^+^) are an important subset of helper T cells which help to the formation of germinal centers and B cells differentiation. In this study, we investigated the proportion and function of Tfh using NUP98-HOXD13 transgenic (NHD13) mice model with MDS phenotype. The proportion of Tfh from bone marrow and spleen of NHD13 mice decreased compared with wild type (WT) mice tested by flow cytometry. In NHD13 mice spleens, there were decreased CXCR5^+^ cells and increased PD-1^+^ cells using immunohistochemistry. The active markers (ICOS, CD40L and OX40) expressed on Tfh of NHD13 mice were decreased. In contrast, PD-1 expression on Tfh of NHD13 mice was higher than that of WT mice. After coculture with Tfh from NHD13 mice, IgG and IgM production of B cells were decreased. In conclusion, the proportion and function of Tfh in the MDS mice model were altered. The dysfunction and reduction of Tfh may inhibit B cells differentiation and antibody production. Abnormal Tfh might contribute to the immune tolerance promoting the progression of MDS.

## INTRODUCTION

Myelodysplastic syndromes (MDS) are a group of heterogeneous malignant clonal diseases of hematopoietic stem cells (HSC), characterized by a normal or hypercellular bone marrow with ineffective and dysplastic hematopoiesis resulting in cytopenia. MDS usually have high mortality, poor prognosis and a lack of specific treatments. Many studies have confirmed that MDS have a high risk of transformation to acute leukemia (AML) due to increased malignant hematopoietic clones (leukemic stem cells, LSC) [1–4].

The pathogenesis of MDS is still unclear. Until now, researchers have demonstrated that altered cytogenetics (including somatic gene mutation, epigenesis, haploinsufficiency) and micro-environments contribute to the pathogenesis and progression of MDS. Abnormal DNA methylation, RNA splicing, or chromatin modification (such as DNMT3A, TET2, SF3B1, EZH2, ASXL1, et al.) participate in the mechanisms of MDS. The gene mutation or molecular abnormalities of HSC induce transformation to LSC [5, 6]. Recently, some studies have proved that abnormal immune systems facilitate the development of MDS and the progression to AML [7–9]. Immune deficiency may lead to immune escape of LSC resulting in malignant clone proliferation [10].

The NUP98-HOXD13 (NHD13) fusion gene, which is constructed with nucleoporin 98KDa (NUP98) amino terminus and human homeobox 13 (HOXD13) fusion gene, occurs in MDS or acute nonlymphocytic leukemia patients. In 2005, Lin YW et al. reported a NHD13 transgenic mouse model for MDS, by mouse vav1 oncogene expression regulatory elements in C57BL/6J WT mice [11]. The transgene was expressed specifically in hematopoietic tissues, including thymus, spleen and bone marrow. NHD13 mice can develop MDS phenotype at an early age, and more than half of these mice progress to AML between 4 and 14 months of age. Thus, NHD13 mice are the most commonly used as MDS mouse models [12–16]. In previous studies, NHD13 mice showed altered hematopoietic stem /progenitor cells (HSPCs), abnormal bone marrow microenvironment (BMME) and immune dysfunction including dysfunctional B cells [17, 18].

Follicular helper T cells (Tfh) are new subsets of CD4+ T lymphocytes, which can induce the proliferation and differentiation of B cells. IL-21, the major cytokine produced by Tfh, can promote B cells to differentiate into plasma cells and induce antibodies production by interacting with its receptors on B cells. The major chemokine receptor of Tfh is CXCR5 [19, 20].

In this study, we investigated the proportion and function of Tfh in NHD13 mice to explore the role of Tfh in B cell dysfunction in MDS. We found that the proportion and function of Tfh were aberrant in MDS mice models which could impair antibody production of B cells.

## RESULTS

### The proportion of Tfh from BM and spleen of NHD13 mice were decreased

**Figure 1:**
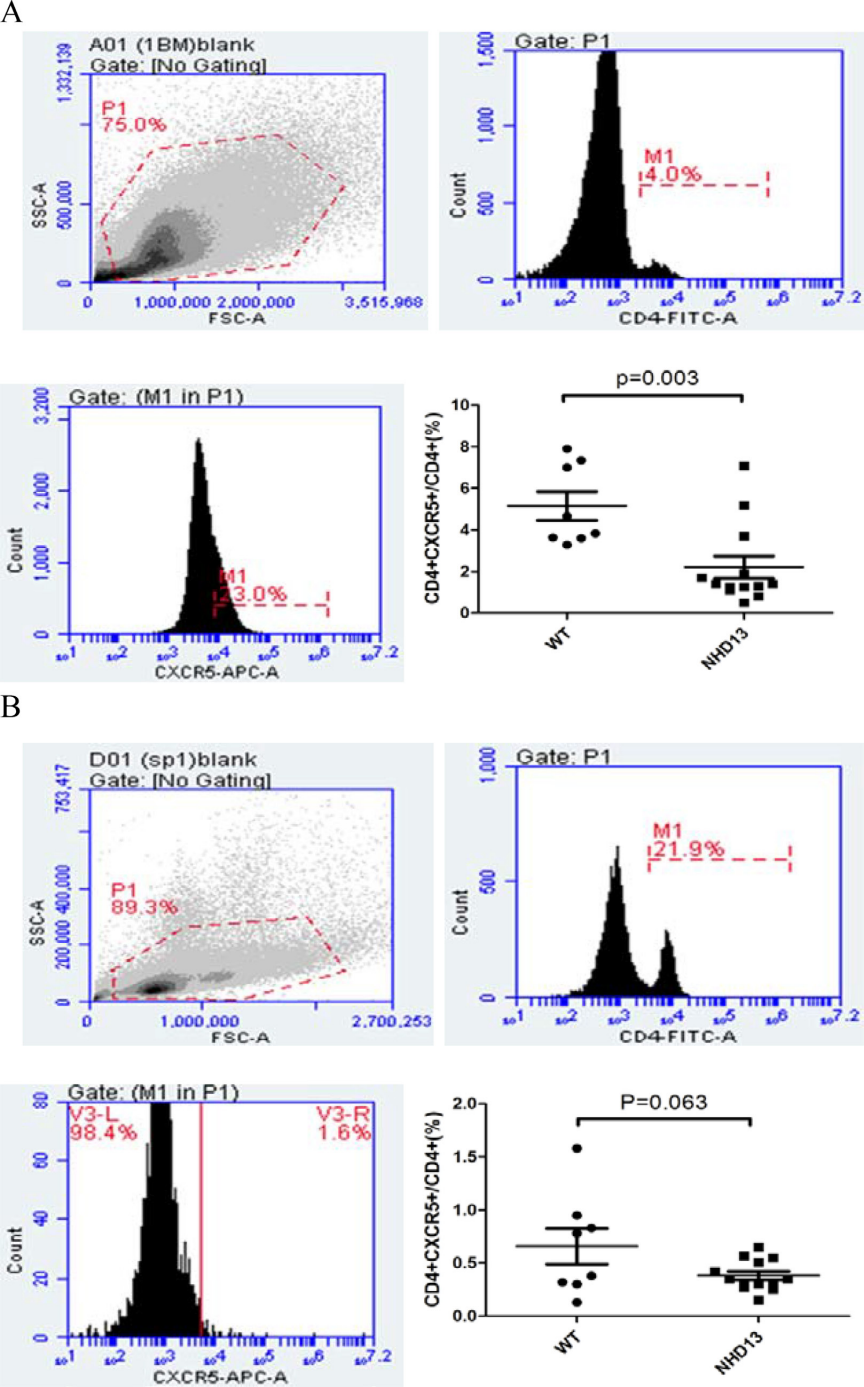
(**A**) The proportion of Tfh in BM of NHD13 mice was lower than that of WT mice (*P* < 0.01). (**B**) The proportion of Tfh in spleen of NHD13 mice was lower than that of WT mice, but the difference had no statistical significance (*P* > 0.05).

The proportion of Tfh (CD4+CXCR5+) from BM of NHD13 mice (2.20 ± 0.54%) was lower than that of WT mice (5.15 ± 0.68%, *P* < 0.01) (Figure 1A). The proportion of Tfh from spleen of NHD13 mice (0.38 ± 0.04%) was lower than that of WT mice (0.66 ± 0.17%). But there was no statistical difference (*P* > 0.05). (Figure 1B).

### The PD-1 expression on Tfh from BM and spleen of NHD13 mice were increased

**Figure 2:**
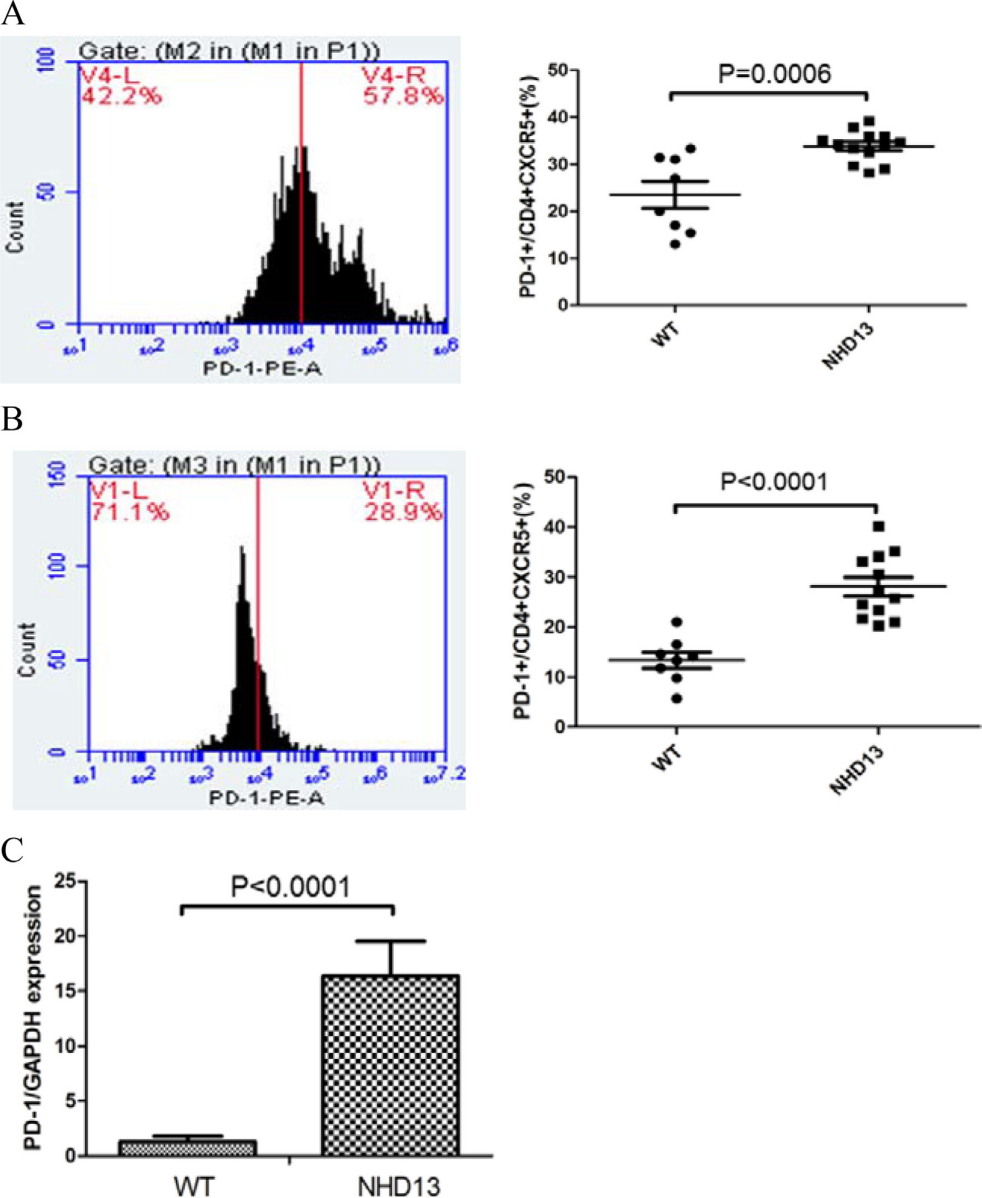
(**A**) PD-1 expression on Tfh from BM of NHD13 mice was higher than that of WT mice (*P* < 0.01). (**B**) PD-1 expression on Tfh from spleen of NHD13 mice was higher than that of WT mice (*P* < 0.01). (**C**) PD-1 mRNA expression in Tfh from spleen of NHD13 mice was higher than that of WT mice (*P* < 0.01).

The PD-1 expression on Tfh from BM of NHD13 mice (33.82 ± 0.91%) was higher than that of WT mice (23.51 ± 2.86%, *P* < 0.01) (Figure 2A). The PD-1 expression on Tfh from spleen of NHD13 mice (28.09 ± 1.86%) was higher than that of WT mice (13.35 ± 1.60%, *P* < 0.01) (Figure 2B). The expression of PD-1 mRNA of Tfh from spleen from NHD13 mice was (16.35 ± 3.17), which was higher than that of WT mice (1.28 ± 0.53%, *P* < 0.01) (Figure 2C).

### The OX40, ICOS and CD40L expression on Tfh of NHD13 mice were decreased, especially in spleen

**Figure 3:**
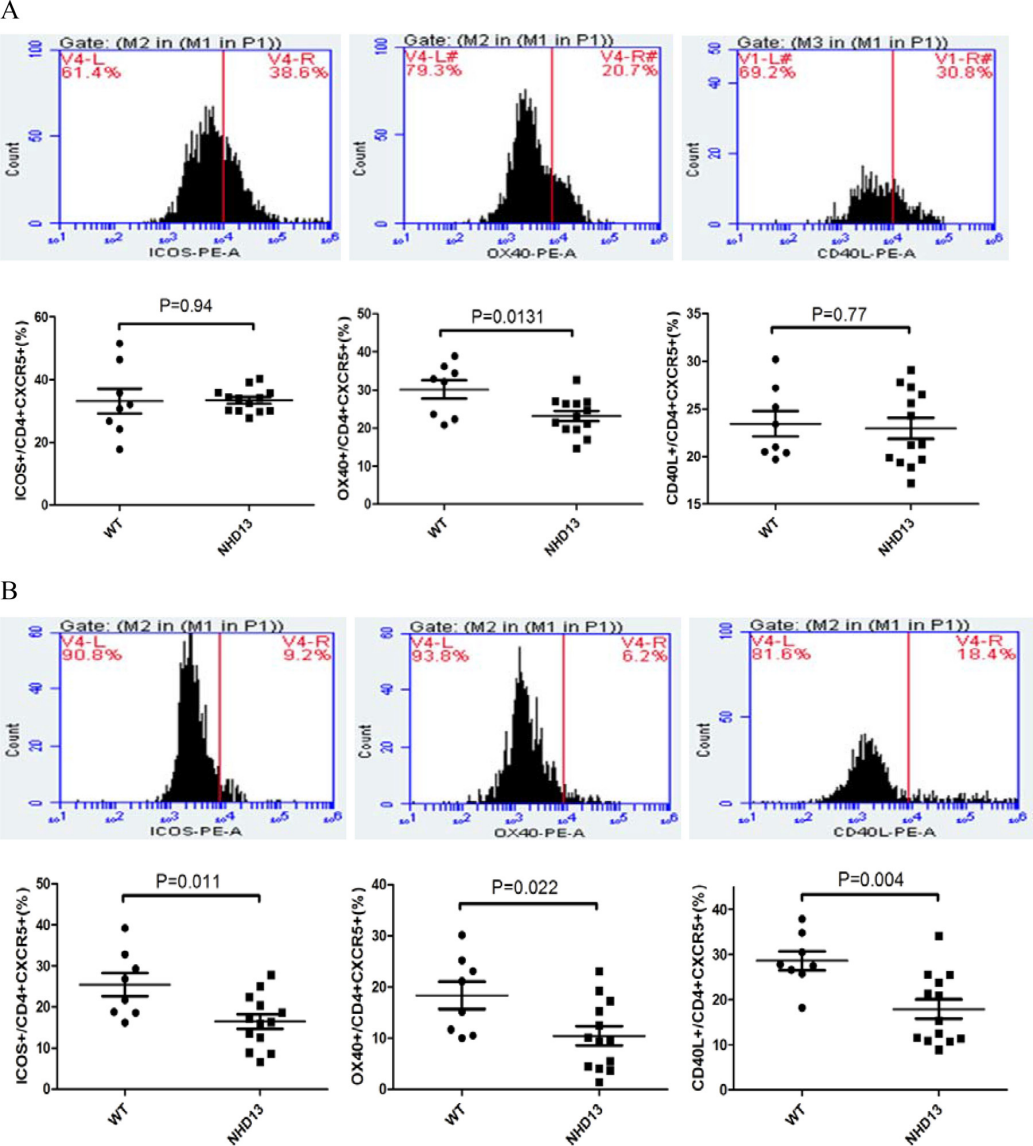
(**A**) The OX40 expression on Tfh from BM of NHD13 mice was lower than that of WT mice (*P* < 0.05). There were no statistical differences in ICOS or CD40L expression between two groups (both *P* > 0.05). (**B**) The expression of OX40, ICOS and CD40L on Tfh from spleen of NHD13 mice were lower than those of WT mice (all *P* < 0.05).

The OX40 expression on Tfh from BM of NHD13 mice (23.15 ± 1.35%) was lower than that of WT mice (30.16 ± 2.45%, *P* < 0.05). The ICOS expression on Tfh from BM of NHD13 mice was (33.42 ± 1.06)%, while that of WT mice was (33.16 ± 3.98)%. The CD40L expression on Tfh from BM of NHD13 mice was (22.94 ± 1.10)%, while that of WT mice was (23.45 ± 1.34)%. There were no statistical differences in ICOS or CD40L expression between the two groups (both *P* > 0.05) (Figure 3A).

The expression of OX40 (10.46 ± 1.87%), ICOS (16.46 ± 1.78%) and CD40L (17.88 ± 2.17%) on Tfh from spleen of NHD13 mice was lower than those of WT mice (18.36 ± 2.45%, 25.43 ± 2.68%, 28.63 ± 2.12%, respectively) (all *P* < 0.05) (Figure 3B).

### CXCR5 expression was lower and PD-1 expression was higher on spleen cells using IHC

**Figure 4:**
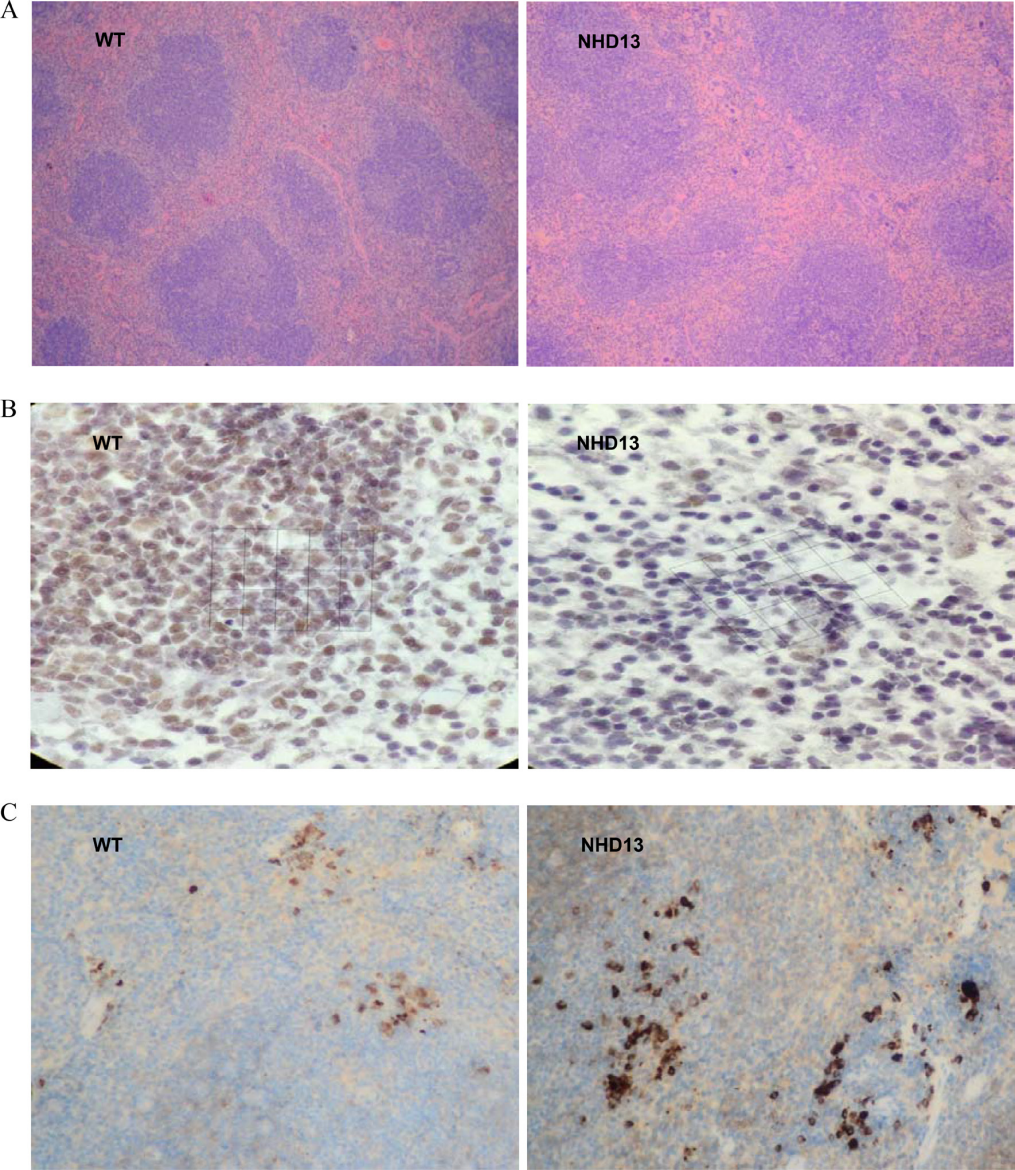
CXCR5 and PD-1 expression were tested by IHC (**A**) In NHD13 mice, the spleen lymphoid follicles and germinal centers were reduced, and red pulps were widened (10×10 visual field), (**B**) CXCR5 expression on spleen cells from NHD13 mice was lower than that of WT mice (10 × 40 visual field), (**C**) PD-1 expression on spleen cells from NHD13 mice was higher than that of WT mice (10×10 visual field).

Spleen lymphoid follicles and germinal centers of NHD13 mice were significantly reduced. Red pulps of NHD13 mice spleen were widened (Figure 4A). IHC results showed that NHD13 mice had lower CXCR5 (Figure 4B) expression and higher PD-1 expression on spleen cells compared with WT mice (Figure 4C).

### Serum IgG and IgM levels of NHD13 mice were decreased

**Figure 5:**
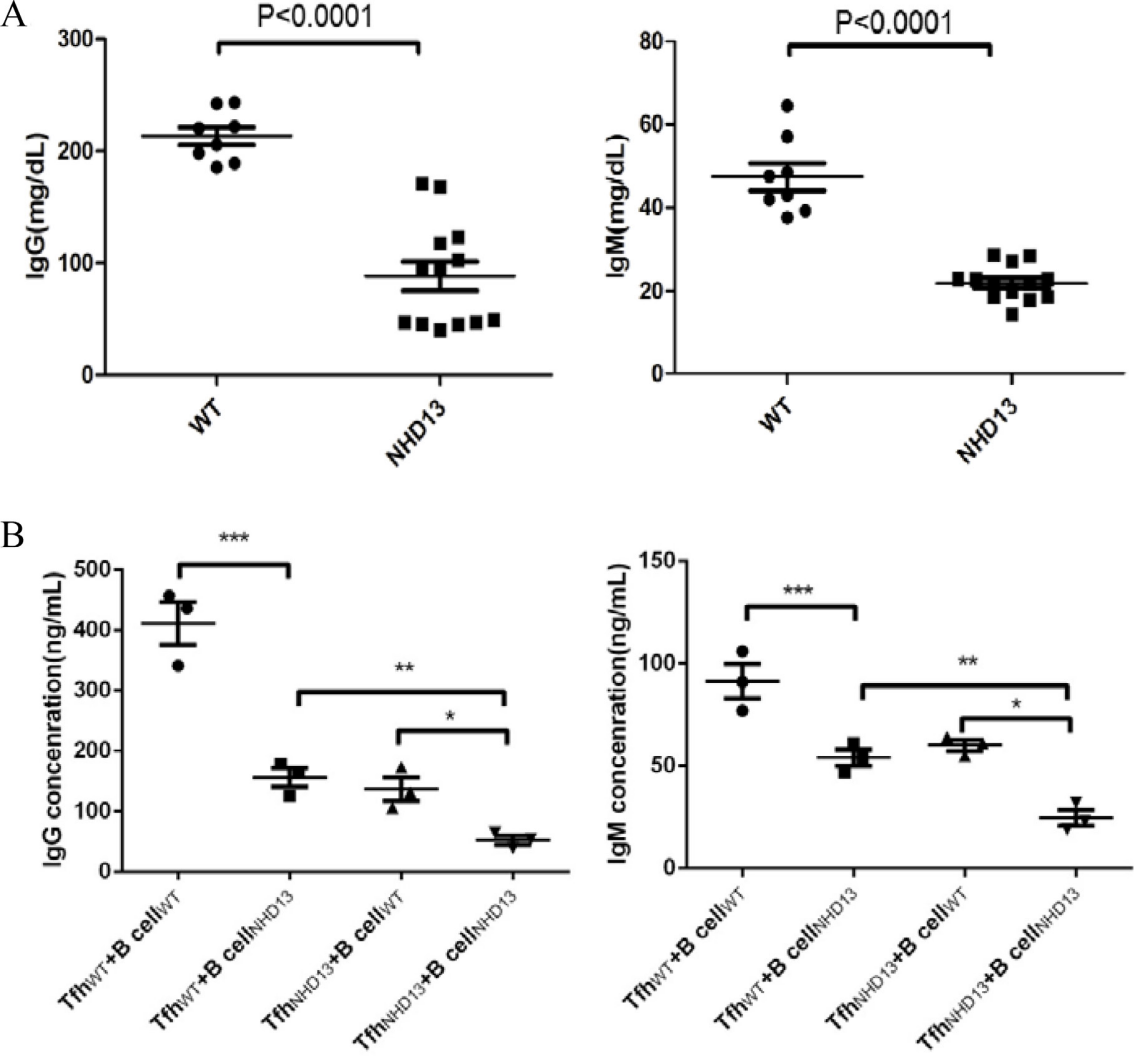
(**A**) The serum IgG and IgM levels of NHD13 mice were decreased compared with that of WT mice (both *P* < 0.01). (**B**) Compared with Tfh_WT_ +B cell_WT_ co-culture, the IgG and IgM production of B cells were decreased after co-cultured with Tfh_NHD13_. Meanwhile, the IgG and IgM production were lowest in co-culture of Tfh_NHD13_ +B cell_NHD13_. (^*^,^**^,^***^ *P* < 0.05).

The serum levels of IgG (88.27 ± 13.03 mg/dL) and IgM (21.88 ± 1.18 mg/dL) of NHD13 mice were decreased compared with those of WT mice (213.30 ± 7.95 mg/dL and 47.39 ± 3.28 mg/dL, respectively, both *P* < 0.01) (Figure 5A).

### The IgG and IgM production of B cells were decreased after co-culture with Tfh _NHD13_

After co-cultured with auto-Tfh (Tfh_WT_), the IgG and IgM production of B cells from WT mice (B cell_WT_) were (411.3 ± 35.69) ng/ml and (91.33 ± 8.37) ng/ml, respectively (*N* = 3). After co-culture with auto-Tfh (Tfh_NHD13_), the IgG and IgM production of B cells from NHD13 mice (B cell_NHD13_) were (52.67 ± 7.54) ng/ml and (24.67 ± 3.84) ng/ml, respectively (*N* = 3). After Tfh_NHD13_ +B cell_WT_ co-culture, the IgG and IgM production of B cell_WT_ were (137.0 ± 19.50) ng/ml and (60.00 ± 2.65) ng/ml, respectively (*N* = 3). After Tfh_WT_ + B cell_NHD13_ co-culture, the IgG and IgM production of B cell_NHD13_ were (156.3 ± 15.62) ng/ml and (54.00 ± 4.04) ng/ml, respectively (*N* = 3). These results indicated that the IgG and IgM production of B cells was decreased after co-culture with Tfh _NHD13_. Furthermore, both Tfh and B cells function were impaired in NHD13 mice (Figure 5B).

## DISCUSSION

MDS are a group of clonal hematopoietic diseases with a high risk of transforming into AML. Recent studies have confirmed that immunological abnormalities play important roles in MDS pathogenesis [1]. Previous studies showed that patients with MDS had aberrant cellular and humoral immunity, including defective T cells and B cells, decreased helper T cells (Th) and increased regulatory T cells (Treg). MDS patients with immune dysfunction cannot clean up malignant clones [7, 9, 21]. Hence, altered immune status in MDS may accelerate LSC survival and amplification, which help to AML transformation.

In NHD13 mouse model, Balderman SR et al. [18] proved that altered bone marrow microenvironments (BMME) may facilitate the progression of MDS. There was accelerated AML transformation and mortality rate in NHD13 compared with WT. And replacement of the MDS BMME with a WT BMME in mice model showed mitigated transformation to leukemia and death in recipients of NHD13 marrow. Though immune cells as the important components of BMME, Choi CW et al. [22] reported that NHD13 mice had lymphopenia with decreased T and B lymphocytes counts. NHD13 mice also had abnormal differentiation from pro-B to pre-B cells. This suggested that impaired differentiation and function in B lymphocytes might promote the development of MDS.

Tfh is the main subset of Th which assistant with differentiation and antibodies production of B cells. We found that NHD13 mice at 6–8 weeks had cytopenia and dysplasia as morphological changes of MDS (Supplementary Figures 2–3). Compared with WT mice, the proportions of Tfh in BM and spleen from NHD13 mice were decreased, especially in BM. The active markers (including ICOS, CD40L and OX40) expressed on Tfh of NHD13 mice were decreased. Meanwhile, NHD13 mice had lower serum IgG and IgM levels compared with WT mice. After co-culture with Tfh from NHD13 mice, IgG and IgM production of B cells was decreased. These results indicated that reduction and dysfunction in Tfh could impair B cells function and humoral immunity in MDS mouse model.

In this study, we measured PD-1 expression on Tfh from NHD13 mice compared with WT mice. We found PD-1 expression on Tfh was significantly increased in NHD13 mice. Thus, we inferred that the reduction of Tfh in MDS might be due to increased PD-1 expression on Tfh.

PD-1 is an immunosuppressive receptor which is expressed on activated T lymphocytes, B lymphocytes and macrophages. PD-L1 is the major ligand of PD-1 expressed on non-lymphoid tissues and activated antigen presenting cells (APC) [23, 24]. PD-1 plays an important role in the establishment of immune tolerance and prevention of autoimmune diseases [25]. In normal conditions, PD-1 could maintain immune tolerance to self-antigens interacting with PD-L1. A combination of PD-1 and PD-L1 contribute to negative regulation in the immune network, which inhibit the proliferation and activation of lymphocyte or the release of inflammatory cytokines [9, 26].

In recent years, researchers have confirmed that PD-L1 was highly expressed on many kinds of tumors cells. PD-L1 interacting with PD-1 on lymphocytes infiltrated in tumor tissues could inhibit anti-tumor response of lymphocytes, leading to immune escape of tumor cells [8, 27]. PD-L1 and/or PD-1 blockades have achieved clinical benefits in cancer treatments. In a clinical trial of advanced tumor cases (including 207 various types), the response rate of PD-L1 blockade treatment was 6–17% [28]. In another clinical trial of BMS-936558 (296 cases of various types of cancer patients), the response rate of advanced non-small cell lung cancer was 18%, 28% of melanoma, and 27% of renal cell carcinoma [29]. In an international multi-center clinical trial of drug-resistant refractory melanoma, PD-1 blockade pembrolizumab induced a 26% response rate with mild side effects [30].

In malignant hematological diseases, the clinical trials of PD-1 blockade (nivolumab) in treatments of refractory and relapsed Hodgkin lymphoma showed that 87% of patients had good response, including 17% with a complete response (CR) rate [31]. Yang H et al. [32] also suggested that PD-1 signaling was involved in MDS pathogenesis and resistance mechanisms to hypomethylating treatments. According to our results, we inferred that increased PD-1 on Tfh might contribute to the reduction and dysfunction of Tfh in MDS. The blockade of the PD-1 pathway might be of benefit to MDS patients.

We also measured the proportion of Tfh and PD-1 expression on Tfh from MDS patient samples. The data showed similar tendency to the results of mice model (Supplementary Table 1, Supplementary Figure 4). But sample size was very limited which need further studies.

In conclusion, the proportion and function of Tfh were abnormal in MDS mice model, which might be due to increased PD-1 expression on Tfh. Altered Tfh could inhibit antibodies production of B cells. Since the molecular mechanisms of impairment of Tfh in MDS have not been fully elucidated, further research is ongoing.

## MATERIALS AND METHODS

### Subjects

Thirteen NHD13 mice (StrainNa: C57BL/6-Tg (Vav-NUP98/HOXD13; Stock Number: 010505) were purchase from Jackson Lab (USA). Eight homological wild type C57BL/6J (WT) mice were used as controls. There were thirteen mice in the experimental group and eight mice in the control group. All mice were male (age range 6 to 8 weeks). The mice were fed in the Animal Center of the Institute of Radiation Medicine, Academy of Medical Science and Peking Union Medical College. All studies were approved by the Tianjin Medical University intramural animal care and use committee.

### The detection of the quantity and programmed death 1 receptor (PD-1) expression on Tfh from mice spleen and bone marrow by flow cytometry (FCM)

The spleen samples were obtained from NHD13 and WT mice, and were stored in 2 ml PBS and gently ground. The cell suspension was acquired after being filtered, centrifuged and after hemolysis (Hemolysin, Becton Dickinson Company, USA). Finally, 1 × 10^6^ cells were immunostained with rat-anti-mouse monoclonal CD4-FITC, CXCR5-APC, PD-1-PE, OX40-PE, ICOS-PE and CD40L-PE (BD company, USA) respectively, and analyzed by FCM (BD Accuri C6, BD company, USA). Meanwhile, BM samples were acquired from the femur of the mice. After being filtered, 1 × 10^6^ cells were stained with CD4-FITC, CXCR5-APC, PD-1-PE, OX40-PE, ICOS-PE and CD40L-PE respectively, and analyzed by FCM. The data was analyzed by BD Accuri C6 software (BD Company, USA).

### Immunohistochemistry (IHC)

The 4–6 μm sections of paraffin embedded tissues were stained with CXCR5 antibody (abcam, USA) or PD-1 antibody (abcam, USA). Deparaffinized tissue sections were pretreated with 3.0% hydrogen peroxide in methanol for at least 15 minutes to block endogenous peroxidase activity. The tissue sections were incubated with the primary antibodies diluted in 1% animal serum PBS-T at room temperature for between and 1 and 2 hours. Subsequently, the tissue sections were incubated with secondary antibody at room temperature for 1 hour following the manufacturer's guidelines for reagent preparation.

### The expression of PD-1 mRNA of sorted Tfh cells from spleen

Tfh (CD4+CXCR5+ cells) from spleen of NHD13 and WT mice were sorted by the FACSAria (BD Biosciences). Total RNA was extracted using Trizol (Takara Bio, CA), and cDNA was generated using reverse transcriptase kit (Takara Bio,CA). Acquired the same amount of cDNA, performed qPCR reaction. The reaction system was 25uL: SYBR^®^Premix Ex Taq II (Takara Bio, China) 12.5ul, forward and reverse primer, each 0.5ul, and sterilized distilled water and samples total 11.5ul. Primers used are listed as follows: PD-1 forward 5′- TTGACAGCAGGGAAGGAAAG -3′ reverse 5′- AGGAGAGCCAGAACCCAACT -3′; GAPDH forward 5′- ACGGCAAATTCAACGGCACAGTCA -3′, reverse 5’- TGGGGGCATCGGCAGAAGG -3′. Applied Bio-Rad CFX Manager software, each group relative quantitative using 2-ΔΔCt values: ΔΔCt = (C_ttarget_−C_tGAPDH_)target−(C_ttarget_−C_tGAPDH_)ctrl.

### Serum and IgG and IgM detected by ELISA

The levels of serum IgG and IgM were measured by ELISA kit according to the manufacturer's instructions (Bethyl Laboratories).

### Tfh and B cells co-culture

Tfh were sorted by the FACSAria (BD Biosciences) (from spleen). B cells (from spleen) were sorted by the MACS kit (Miltenyi Biotec). Sorted cell purity was > 90% (Supplementary Figure 1). Tfh and B cells (25 000 cells per well, 1:1 ratio) were co-cultured in RPMI 1640 medium (containing L-glutamine, penicillin, streptomycin, 10% fetal bovine serum, and 1 mg/mL staphylococcal enterotoxin (SEB; Toxin Technology)) for 7 days. IgG and IgM were measured by ELISA kit (Bethyl Laboratories).

### Statistical analysis

All statistical analyses were performed with SPSS 20.0 software (SPSS Science). The data is presented as mean ± SEM. For normal distribution data between two independent groups, Student's *t*-test was used. For skewed distribution data, Wilcoxon test was used. A value of *P* < 0.05 was considered to be statistically significant.

## SUPPLEMENTARY MATERIALS FIGURES AND TABLE


