# Supplementary material for: Altered follicular helper T cell impaired antibody production in a murine model of myelodysplastic syndromes
Source: Oncotarget. 2017 Oct 6;8(58):98270–9. doi: 10.18632/oncotarget.21548 (PMC5716728; doi:10.18632/oncotarget.21548)
Supplement: Supplementary file 1 [file oncotarget-08-98270-s001.pdf]

Altered follicular helper T cell impaired antibody production in a murine model of myelodysplastic syndromes

SUPPLEMENTARY MATERIALS

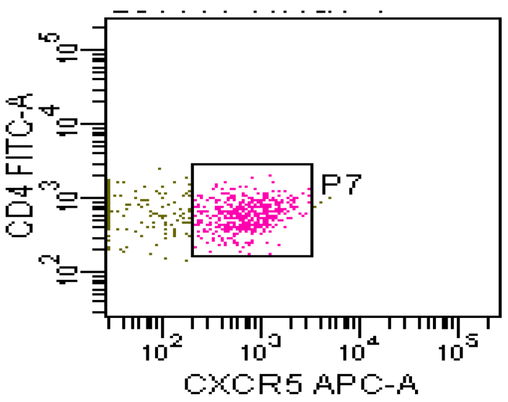

Supplementary Figure 1: The purity of sorted Tfh tested by FCM (> 90%).

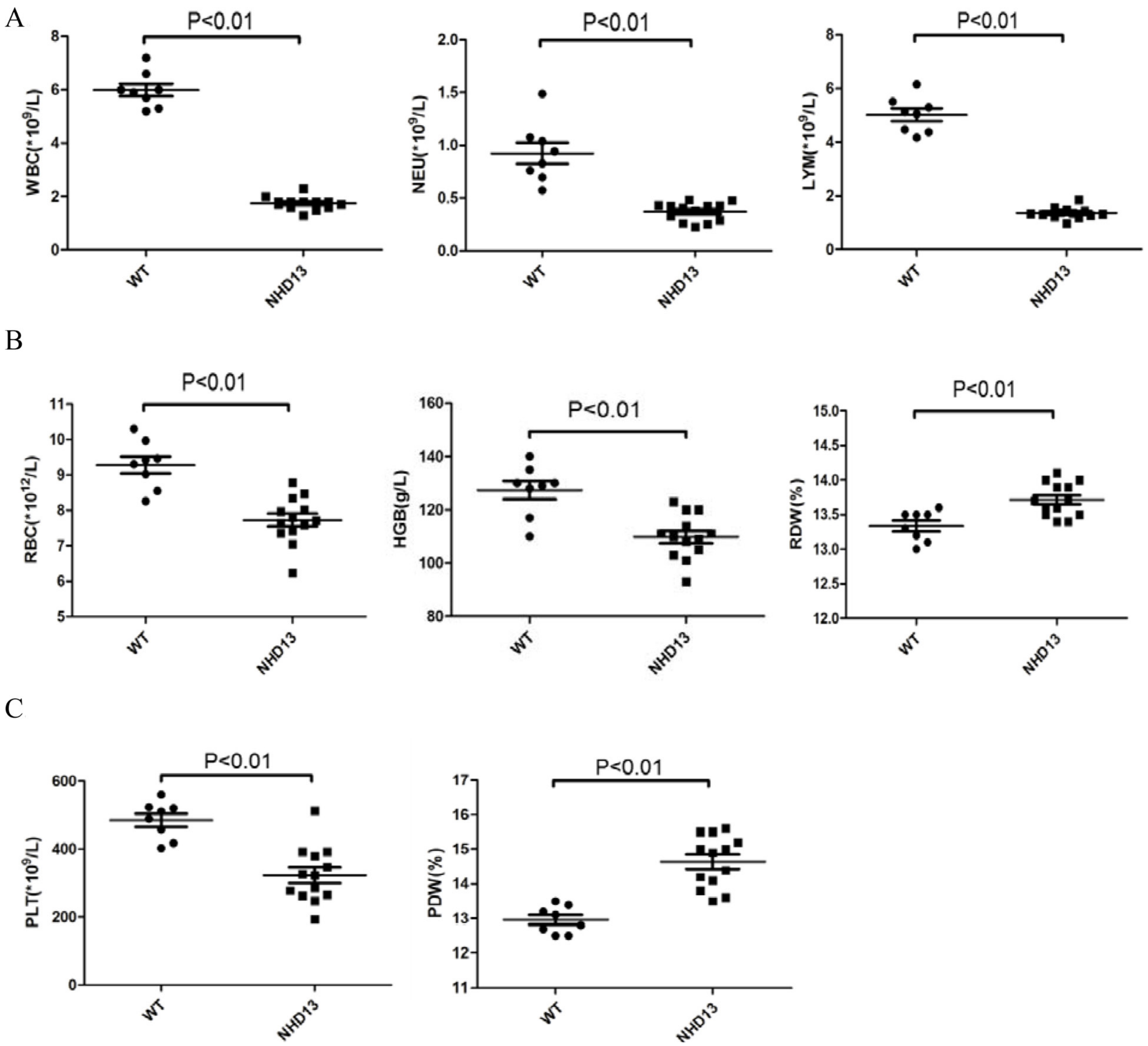

Supplementary Figure 2: Whole blood samples anticoagulated by EDTA were collected from the angular vein of mice, and analyzed by Celltac E Automatic Blood cell analyzer MEK-722 (Photoelectricity Joint-stock company, Japan). Complete blood counts of NHD13 mice were decreased compared with WT mice, (**A**) The counts of white blood cells (WBC), neutrophils (NEU) and lymphocytes (LYM) of NHD13 mice ((1.746 ± 0.067) × 10^9/L, (0.370 ± 0.024) × 10^9/L and (1.361 ± 0.057) × 10^9/L, respectively) were lower than those of WT mice ((5.988 ± 0.233) × 10^9/L, (0.925 ± 0.101) × 10^9/L and (5.020 ± 0.234) × 10^9/L, respectively) (all $P < 0.01$), (**B**) The red blood cells (RBC) counts and hemoglobin (Hb) levels of NHD13 mice ((7.729 ± 0.181) × 10^{12}/L and (109.8 ± 2.3) g/L, respectively) were lower than those of WT mice ((9.285 ± 0.239) × 10^{12}/L and (127.4 ± 3.4) g/L, respectively) (both $P < 0.01$). The red cell distribution width (RDW) of NHD13 mice (13.72 ± 0.07%) was higher than that of WT mice (13.34 ± 0.08%, $P < 0.01$). (**C**) The PLT counts of NHD13 mice ((323.3 ± 23.0) × 10^9/L) were lower than that of WT mice ((484.9 ± 19.5) × 10^9/L, $P < 0.01$). The platelet distribution width (PDW) of NHD13 mice (14.64 ± 0.21%) was higher than that of WT mice (12.96 ± 0.14%, $P < 0.01$).

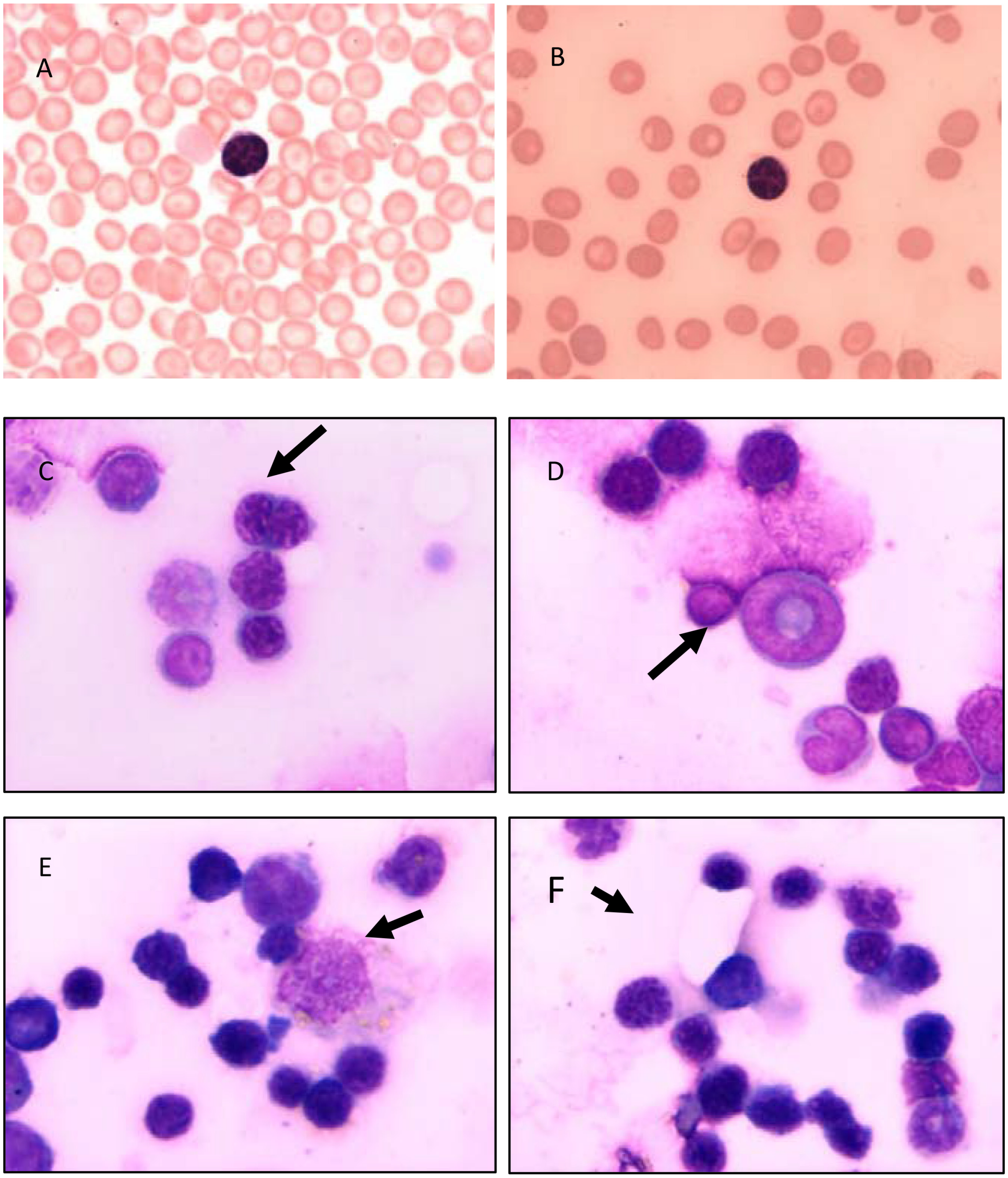

Supplementary Figure 3: Peripheral blood samples from the angular vein and bone marrow samples from the femur of mice were spotted onto the smears. NHD13 mice had cytopenia and dysplasia observed through peripheral blood and BM smears. (**A**) Normal blood smear of WT mouse, (**B**) cytopenia in blood smear of NHD13 mouse, (**C**) bi-nucleated erythrocyte in blood smear of NHD13 mouse, (**D**) ringed nucleated neutrophil in blood smear of NHD13 mouse, (**E–F**) erythroblastic island in BM smear of NHD13 mouse (Wright stain, 10 × 100 visual field).

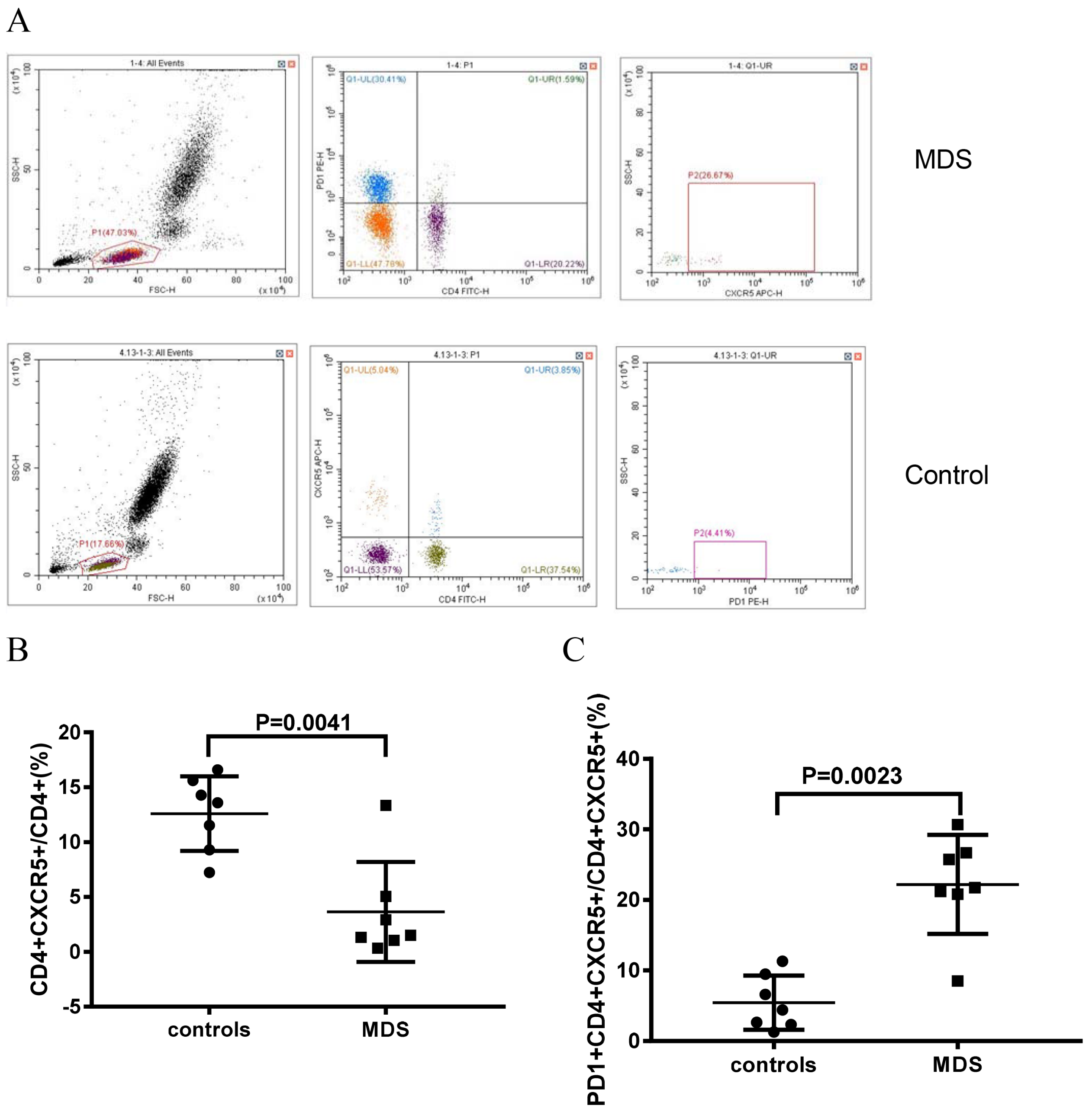

Supplementary Figure 4: (**A**) The proportion of Tfh and PD-1 expression on Tfh in peripheral blood from MDS patients and normal controls were tested by FCM. All samples were stained with CXCR5-APC, CD4-FITC, PD1-PE and the mouse isotype controls (BD, USA). Finally, 20000 cells were acquired on a FACS-Calibur flow cytometry (BD Biosciences, USA) and analyzed by CellQuest software version 3.1.(**B**) The proportion of Tfh from MDS patients (3.65 ± 1.72%) was lower than that of normal controls (12.59 ± 1.39%, $P < 0.01$). (**C**)The PD-1 expression on Tfh from MDS patients (22.20 ± 2.56%) was higher than that of normal controls (5.44 ± 1.45%, $P < 0.01$).

Supplementary Table 1: The clinical characteristics of the MDS patients and the Controls

	Number	Median age	classification
MDS patients*	7<br>(Male = 5,<br>Female = 2)	60<br>(range 44–72 years)	MDS with single lineage dysplasia (MDS-SLD, *n* = 2);<br>MDS with multilineage dysplasia (MDS-MLD, *n* = 2);<br>MDS with excess blasts (MDS-EB-1, *n* = 1);<br>MDS with excess blasts (MDS-EB-2, *n* = 2)
Healthy controls	7<br>(Male = 3,<br>Female = 4)	47<br>(range 28–69 years)	

*All patients were newly diagnosed in the Department of Hematology, General Hospital of Tianjin Medical University from March 2017 to July 2017, according to definitions and standards in the diagnosis of the myelodysplastic syndromes in 2016 [33] . The study was approved by the Ethics Committee of Tianjin Medical University General Hospital. All patients and normal individuals in accordance with the Declaration of Helsinki.
